# Capsaicin Displays Anti-Proliferative Activity against Human Small Cell Lung Cancer in Cell Culture and Nude Mice Models via the E2F Pathway

**DOI:** 10.1371/journal.pone.0010243

**Published:** 2010-04-20

**Authors:** Kathleen C. Brown, Ted R. Witte, W. Elaine Hardman, Haitao Luo, Yi C. Chen, A. Betts Carpenter, Jamie K. Lau, Piyali Dasgupta

**Affiliations:** 1 Department of Pharmacology, Physiology and Toxicology, Joan C. Edwards School of Medicine, Marshall University, Huntington, West Virginia, United States of America; 2 Department of Biochemistry and Microbiology, Joan C. Edwards School of Medicine, Marshall University, Huntington, West Virginia, United States of America; 3 Department of Biology, Alderson-Broaddus College, Phillipi, West Virginia, United States of America; 4 Department of Anatomy and Pathology, Joan C. Edwards School of Medicine, Marshall University, Huntington, West Virginia, United States of America; University of Hong Kong, Hong Kong

## Abstract

**Background:**

Small cell lung cancer (SCLC) is characterized by rapid progression and low survival rates. Therefore, novel therapeutic agents are urgently needed for this disease. Capsaicin, the active ingredient of chilli peppers, displays anti-proliferative activity in prostate and epidermoid cancer *in vitro*. However, the anti-proliferative activity of capsaicin has not been studied in human SCLCs. The present manuscript fills this void of knowledge and explores the anti-proliferative effect of capsaicin in SCLC *in vitro* and *in vivo*.

**Methodology/Principal Findings:**

BrdU assays and PCNA ELISAs showed that capsaicin displays robust anti-proliferative activity in four human SCLC cell lines. Furthermore, capsaicin potently suppressed the growth of H69 human SCLC tumors *in vivo* as ascertained by CAM assays and nude mice models. The second part of our study attempted to provide insight into molecular mechanisms underlying the anti-proliferative activity of capsaicin. We found that the anti-proliferative activity of capsaicin is correlated with a decrease in the expression of E2F-responsive proliferative genes like cyclin E, thymidylate synthase, cdc25A and cdc6, both at mRNA and protein levels. The transcription factor E2F4 mediated the anti-proliferative activity of capsaicin. Ablation of E2F4 levels by siRNA methodology suppressed capsaicin-induced G1 arrest. ChIP assays demonstrated that capsaicin caused the recruitment of E2F4 and p130 on E2F-responsive proliferative promoters, thereby inhibiting cell proliferation.

**Conclusions/Significance:**

Our findings suggest that the anti-proliferative effects of capsaicin could be useful in the therapy of human SCLCs.

## Introduction

Small cell lung cancer (SCLC) is an aggressive malignancy representing 13% of all lung cancer cases, with an overall 5-year survival rate of less than 5% [1], [2]. Such statistics emphasize the need for novel treatment strategies for this disease. Recent advances in the basic understanding of molecular events involved in SCLC progression have led to the identification of potential agents for therapeutic interventions [2], [3], [4], [5]. These strategies include growth factor/receptor-specific inhibitors, protein kinase inhibitors and nutritional agents. The identification of nutritional agents that display anti-proliferative activity may represent a novel therapeutic avenue in human SCLC.

Capsaicin, the major active ingredient of chili peppers, is used topically to treat pain and inflammation associated with a variety of diseases [6], [7]. Chemoprevention studies demonstrate that capsaicin can suppress carcinogenesis of the skin, colon, lung, tongue and prostate [8], [9], [10], [11], [12]. Although these studies have addressed the chemopreventative potential of capsaicin, only a few have addressed its potential as an anti-cancer agent. For example, capsaicin has been shown to induce apoptosis in non-small cell lung cancer (NSCLC), T-cell leukemia, esophageal carcinoma, astroglioma, prostate, colon and gastric cancer cells in cell culture models [13], [14], [15], [16], [17]. Additionally, the administration of capsaicin has been shown to suppress prostate cancer tumor growth in nude mice models [10], [18].

Apart from causing apoptosis, capsaicin has been found to induce cell cycle arrest in human cancer cells. Several convergent studies have shown that capsaicin-induced G1 arrest in CE 81T/VGH human epidermoid carcinoma cells and prostate cancer cells occur via induction of p53 and the cyclin-dependent kinase (cdk) inhibitor p21 [10], [17], [19], [20], [21]. The treatment of HL-60 human leukemic cells with capsaicin caused G1 arrest via inhibition of cdk2 activity. The anti-angiogenic activity of capsaicin is attributed to its ability to cause G1 arrest in endothelial cells. Capsaicin-induced G1 arrest is correlated with the suppression of cyclin D1 levels, inhibition of cdk4 activity and Rb phosphorylation in endothelial and breast cancer cells [21], [22]. These data raise the possibility that the anti-proliferative activity of capsaicin is mediated by its effects on the E2F-Rb pathway.

The E2F family of transcription factors, consisting of eight member genes (E2F1-E2F8), plays a pivotal role in regulating cell cycle progression and cell proliferation [23], [24], [25], [26]. These E2Fs have been further subclassified into two groups based on their transcriptional regulatory properties on gene promoters. E2F1, E2F2 and E2F3 are often referred to as “activator” E2Fs because they transcriptionally activate E2F target proliferative genes such as cyclin E, cdc25A and cdc6 [25], [27], [28], [29]. These target genes then induce the entry of cells into S-phase, thereby promoting cell cycle progression. The second subclass, E2F4 and E2F5, are referred to as the “repressor” E2Fs because they repress the transcription of E2F target proliferative genes. E2F family members E2F6, E2F7 and E2F8 are also repressors. E2F7 and E2F8 are the most recently identified members of this family, and much less is known about their function and regulation [25]. Despite the fact that all transcriptionally active E2Fs bind to the same DNA recognition site on target promoters, they have different functional roles in the cell [26], [29].

Studies in recent years have shown that the activity of E2Fs is stringently regulated by the pocket protein family, namely Rb, p130 and p107. E2Fs 1–3 can bind to the Rb protein, but E2Fs 4 and 5 preferentially bind to p107 and p130 proteins [24], [25], [26], [29], [30]. The Rb family proteins bind to a moiety within the transcriptional activation region of E2Fs, effectively repressing their activity. Thus, quiescent cells contain high levels of E2F proteins bound to Rb, p130 and p107. The onset of mitogenic stimuli causes phosphorylation of Rb, p130 and p107 by cyclin D and E and their associated kinases. The phosphorylation of Rb, p107 and p130 results in their inactivation and dissociation from E2F proteins [25]. These free E2Fs subsequently bind to target proliferative promoters like cyclin E, thymidylate synthase (TS), cdc25A and cdc6, which regulate cell cycle progression [31], [32]. Of these, E2F1–3 stimulate the transcription of the aforementioned genes, whereas E2F4 and E2F5 repress transcription [25]. A majority of human SCLC are deficient in the Rb protein [3]. Therefore, it is probable that the other two pocket proteins, p107 and p130, play a vital role in E2F regulation and cell proliferation in human SCLCs.

The anti-proliferative activity of capsaicin has not been studied in human SCLCs. The present manuscript fills this gap of knowledge and shows for the first time that capsaicin can potently inhibit the proliferation of human SCLCs using multiple cell culture and *in vivo* models. Previous studies have shown that a majority of human SCLCs have mutations in Rb and p53, as well as a dysregulation in the E2F-Rb pathway [3], [4], [5]. Data obtained from microarrays, tissue samples from lung cancer patients and human lung cancer cell lines indicate that cell cycle regulatory molecules, like E2F1–5, p130, Skp2, cyclin D1 and p16, contribute to the growth and progression of lung tumors [33]. Therefore, we hypothesized that capsaicin displays anti-proliferative activity in human SCLCs by regulating the activity of the E2F family of transcription factors.

The present manuscript is an initial study aimed at investigating the anti-proliferative activity of capsaicin in human SCLC in both cell culture and animal models. Here, we demonstrate for the first time that capsaicin displays potent anti-proliferative activity in four human SCLC cell lines *in vitro*. Furthermore, our results show that capsaicin suppresses the growth of human SCLC tumors in CAM and nude mice models. We have also explored the contribution of the E2F family of transcription factors in the growth-inhibitory effects of capsaicin in SCLC cells. We observed that capsaicin inhibits the proliferation of human SCLCs via a specific member of the E2F family, namely E2F4. Ablation of E2F4 levels by two independent siRNA was found to reverse the anti-proliferative effect of capsaicin. Furthermore, capsaicin-induced growth arrest was associated with a decrease in the expression of the E2F target genes cyclin E, TS, cdc25A and cdc6. Finally, chromatin IP (ChIP) analysis of human SCLC cells demonstrated that treatment of capsaicin decreased the recruitment of activator E2Fs, namely E2F2 and E2F3, to proliferative promoters like cyclin E, TS, cdc25A and cdc6. On the other hand, the recruitment of repressor E2F4 was enhanced by capsaicin treatment. Taken together, our data suggest that capsaicin displays potent anti-proliferative activity in human SCLC cells by differentially regulating the E2F family of transcription factors. Furthermore, our results raise the possibility that nutritional agents like capsaicin may be useful for the therapy of human SCLC.

## Materials and Methods

### Ethics Statement

Nude mice were obtained from Charles River Laboratories and acclimatized for one week. They were housed in autoclaved cages with *ad libitum* access to food and water in HEPA-filtered racks and closely monitored by animal facility staff. All procedures involving nude mice were conducted according to the Animal Care and Use guidelines in a facility accredited by the Association for Assessment and Accreditation of Laboratory Animal Care (AAALAC) International and were approved by the Institutional Animal Care and Use Committee (IACUC) of Joan C. Edwards School of Medicine, Marshall University (protocol#371).

### Cell Culture and Transfection

The human SCLC cell lines NCI-H69, NCI-H82 (hereafter referred to as H69 and H82, respectively), DMS53 and DMS114 were obtained from American Type Culture Collection, Rockville, MD. These cell lines were chosen because they have been extensively studied, and their physical and molecular characteristics closely resemble SCLC in patients. Gazdar et al., (1985) originally isolated and characterized the H69 and H82 cell lines [34], [35]. They found that the morphology and growth characteristics of H69 and H82 cells were typical of SCLC tumor cells found in patients. Furthermore, the biochemical profile of these cells (presence of L-dopa decarboxylase, neuroendocrine markers, bombesin-like immunoreactivity, neuron-specific enolase and high concentrations of brain isoenzyme of creatine kinase) was found to be identical to human SCLC tumors observed in patients. Similarly, DMS53 and DMS114 cells were first isolated from human SCLC biopsies and characterized by Pettengill et al., (1980). They found that both DMS53 and DMS114 retained the physical, morphological and biochemical profile of human SCLC tumors observed in the clinic [36].

H69 and H82 were maintained in RPMI-1640 supplemented with 2 mM glutamine, 100 units/ml penicillin, 50 µg/ml streptomycin and 10% fetal bovine serum (FBS). DMS53 human SCLC cells were cultured in Waymouth's MB752/1 media containing 2 mM glutamine, 100 units/ml penicillin, 50 µg/ml streptomycin and 10% FBS. The culture media for DMS114 human SCLC cells was identical to DMS53, except that the media contained an additional 2% sodium bicarbonate.

Primary normal human bronchial epithelial cells (NHBE) and small airway epithelial cells (SAEC) were obtained from Lonza Technologies, Switzerland. NHBEs were maintained in BEBM media containing growth factors. Similarly SAECs were maintained in SABM media supplemented with growth factors. Both BEMB and SABM media were prepared according to the manufacturer's instructions. All experiments using NHBEs and SAECs were performed between passages 3–8 [31].

### MTT Assay

MTT assays were performed as described by Heo et al., (1990). H69 and H82 cells were plated in 96-well plates at a density of 50,000 cells/well. DMS53 and DMS114 cells were plated in 96-well plates at a density of 5,000 cells/well. The plates were incubated for 24 hours to allow complete reattachment of the cells. Subsequently, cells were treated with 50 µM capsaicin for 24 hours, 48 hours or 72 hours. After the indicated time points, 50 µl of MTT solution (5 mg/ml) was added to each well, and the plates were incubated for 4 hours at 37°C [37]. Then, the media was aspirated, and 150 µl of DMSO was added to each well to solubilize the formazan crystals. The absorbance of the plates was measured on an ELISA reader (Benchmark, BioRad) at a wavelength of 540 nm. Each sample was performed in triplicate, and the entire experiment was repeated twice.

### BrdU and PCNA Proliferation Assays

Bromodeoxyuridine (BrdU) labeling enzyme linked immunosorbent assay (ELISA) kits were obtained from Roche Biochemicals and were used to examine the effects of capsaicin on the proliferation of four human SCLC cell lines, H69, H82, DMS53 and DMS114. BrdU is a thymidine nucleotide analog that is incorporated during S-phase (instead of thymidine) only in the DNA of proliferating cells [31], [32], [38].

H69 and H82 cells were plated in 96-well plates at a density of 50,000 cells/well. DMS53, DMS114, SAEC and NHBE cells were plated in 96-well plates at a density of 10,000 cells/well. DMS53 and DMS114 were incubated with serum-free media for 36 hours to remove the effect of endogenous growth factors. After 36 hours, these cells were then re-stimulated with 10% FBS in the presence or absence of indicated concentrations of capsaicin for 18 hours, which is the time required for S-phase entry [31], [32], [38]. The NHBEs and SAECs were rendered quiescent in basal media containing ¼ the amount (v/v) of growth factors for 24 hours (cit). Subsequently, the cells were stimulated with complete media containing the full volume of growth factors for 18 hours as described previously. The rate of BrdU incorporation was measured by ELISA technique, and the percentage of cells in S-phase quantitated by colorimetric evaluation (λ = 405 nm). The absorbance of cells treated with 10% FBS or complete media was assumed to be 100%, and capsaicin-induced decreases in S-phase were calculated as a percentage of the control FBS treated cells. Each sample was tested in triplicate, and the assay was repeated twice.

Cell proliferation was also assessed by measuring the levels of proliferating cell nuclear antigen (PCNA) using a PCNA ELISA kit from Calbiochem. H69, H82, DMS53 and DMS114 were plated in 96-well plates and treated in an identical manner as the BrdU assay described above. Subsequently, the media was removed, and re-suspension buffer (50 mM Tris, pH 8, 5 mM EDTA, 0.2 mM PMSF, 1 µg/ml pepstatin, 0.5 µg/ml leupeptin) was added to the cells. The level of PCNA in the cells was quantitated by measuring the absorbance at 405 nm, according to the manufacturer's protocol. Each sample was tested in duplicate, and the assay was repeated twice for each cell type.

### Cell Cycle Analysis

H69 SCLC cells were used for cell cycle analysis, using a modification of the propidium iodide technique [15], [39]. Briefly, 5×10^5^ cells were used per sample. Each sample was incubated with serum-free media for 36 hours to remove the effect of endogenous growth factors. After 36 hours, the cells were then re-stimulated with 10% FBS in the presence or absence of 50 µM capsaicin for 18 hours, which is the time required for S-phase entry [30]. Cells were harvested, washed twice in buffer (1 mM EDTA in DPBS without calcium and magnesium, supplemented with 1% ultra low IgG FBS, Invitrogen Corporation), fixed in ice cold 70% ethanol and re-suspended in propidium iodine staining solution (50 µg/ml propidium iodide, 25 µg/ml RNase A in DPBS/EDTA buffer) for 30 minutes at 37°C. The samples were analyzed by a BD FACS Aria II flow cytometer (BD BioSciences).

### Lysates and Western Blotting

Lysates for each cell line were made using the NP-40-based lysis protocol [31], [38], [40]. DMS114 cells were grown in 100 cm dishes to approximately 70% confluence. The cells were rendered quiescent by incubating in serum-free media for 36 hours. Subsequently, the cells were re-stimulated with 10% FBS in the presence or absence of indicated doses of capsaicin for 18 hours. Cells were harvested and washed three times with ice cold PBS. Cells were then lysed with M2 lysis buffer (20 mM Tris, pH 7.6, 0.5% NP-40, 250 mM NaCl, 3 mM EGTA, 3 mM EDTA, 4 µM DTT, 5 mM PMSF, 1 mM sodium fluoride, 1 mM sodium orthovanadate, 25 µg/ml leupeptin, 5 µg/ml pepstatin, 5 µg/ml aprotinin, 25 µg/ml trypsin-chymotrypsin inhibitor). Seventy microliters of lysis buffer was added for every 20 µl of packed cell volume. The lysate was rotated at 4°C for 30 minutes and subsequently spun at 15000 g for 15 minutes at 4°C. The supernatant was collected for further analysis. The protein concentration of the lysate was measured using a Bradford Reagent (Bio-Rad Labs). One hundred fifty microgram aliquot of the protein was run on a 10% SDS-PAGE gel and transferred onto nitrocellulose membranes (BioRad Labs).

The relative expression of the indicated proteins was analyzed by western blotting. E2F1 monoclonal and polyclonal E2F2–6 antibodies were obtained from Santa Cruz Biotechnology. Monoclonal antibodies to TS, cdc25A and cdc6 were also obtained from Santa Cruz Biotechnology. Monoclonal antibody to cyclin E was obtained from BD Biosciences. Monoclonal β-actin antibody was obtained from Sigma Chemical Company, USA. Polyclonal GAPDH antibody was obtained from Trevigen, Inc. The secondary antibodies were obtained from Pierce Biotechnologies. The signal obtained in the western blot experiments was detected by the SuperSignal West Dura Extended Duration Substrate (Pierce Biotechnologies). The results of the western blotting assays were quantitated by densitometric analysis (BioRad Gel Documentation System) using the analysis software Quantity 4.5.2.

### Chicken Embryo Chorioallantoic Membrane (CAM) Assay

Specific pathogen-free (SPF) fertile chicken eggs (Charles River Laboratories, North Franklin, CT) were incubated at 37.5°C with 75% relative humidity, and continuously rotated slowly by an automatic egg turner (G.Q.F. Manufacturing Company, Savannah, GA). At Day 9, eggs were candled and windows opened on the shell to expose the CAM [41]. H69 cells (3×10^6^) were suspended in 100 µl cold serum-free medium, mixed with 100 µl cold BD Matrigel Matrix (BD Biosciences, San Jose, CA) and 50 µM capsaicin. These cells were applied to the CAM of each chicken embryo. Eggs were incubated at 37°C for 4 days before tumor implants were removed, photographed and weighed. A total of 12 eggs were assayed for each group [41].

### Antitumor Studies in Nude Mice

Eight 4-week-old male nude mice were obtained from Charles River Laboratories and acclimatized for one week. They were housed in autoclaved cages with *ad libitum* access to food and water in HEPA-filtered racks and closely monitored by animal facility staff. All procedures were conducted according to the Animal Care and Use guidelines in a facility accredited by the Association for Assessment and Accreditation of Laboratory Animal Care (AAALAC) International and were approved by the Institutional Animal Care and Use Committee (IACUC) of Joan C. Edwards School of Medicine, Marshall University (protocol#371).

H69 cells were harvested and re-suspended in a 1∶1 (v/v) solution of serum-free media and Matrigel matrix (BD Biosciences). Two million cells in 100 µL were injected subcutaneously between the scapulae of each mouse [42]. After the tumors reached 100 mm^3^, the mice were switched to control AIN-76A based diet containing 10% corn oil until the tumors reached 800 mm^3^. Subsequently, the mice were divided into two groups. The treatment group (N = 4) was changed to a diet containing 50 mg capsaicin/kg food (which is about 10 mg capsaicin/kg body weight of mouse per day). The control group (N = 4) was continued on the control diet. Mice were weighed once per week. Their food consumption was monitored by weighing the leftover food once per week. The administration of capsaicin caused no discomfort or weight loss in mice. Additionally, food intake was similar between control and capsaicin-treated mice.

The drug treatment was continued until tumors of the control group reached 2000 mm^3^. Tumor lengths (l), widths (w) and height (h) were measured daily (for 6 days out of a week) for each mouse. Tumor volumes were calculated as (l x w x h)/2 [43], [44]. After euthanizing the mice, the tumors were excised. Half of the tumor was snap frozen in liquid nitrogen and used to make lysates. Tumor lysates were prepared using T-Per lysis buffer (Pierce Biotechnology), according to manufacturer's protocol [38]. The other half of the tumor was fixed in formalin and used for immunohistochemistry.

### Caspase Cleavage Assay

Caspase cleavage assay was performed with tumor lysates prepared from control mice and capsaicin-treated mice using the caspase-3 cleavage kit (Chemicon, Temecula). Tumor lysates were prepared using T-Per lysis buffer as described above. An aliquot of 60 µl lysate was used for each reaction. Additionally, 60 µl of cisplatin-treated H69 lysate (made out of treating H69 cells with 30 µM of cisplatin for 72 hours) was used as the positive control, according to manufacturer's protocol. Each sample was tested in duplicate, and the assay was repeated twice for each cell type.

### Immunohistochemistry

Immunostaining was performed using the M.O.M. staining kit (Vector Laboratories, Burlingame, CA, USA). Paraffin-embedded H69 xenograft mouse tissue sections (4 µm) were dewaxed in xylene and subsequently rehydrated in ethanol. Sections were subjected to antigen retrieval treatment using the Antigen Retrival kit (BioGenex Inc.), according to the manufacturer's protocol. The sections were then treated with Proteinase K treatment (20 µg/ml) for 15 minutes and quenched of endogenous peroxidases in 0.3% H_2_O_2_ solution in methanol for 30 minutes. The sections were blocked using the Avidin-Biotin blocking kit (Vector Laboratories, Burlingame, CA, USA). The sections were incubated with anti-PCNA (BioGenex Inc.) monoclonal primary antibody (1∶100 dilution) for one hour at room temperature. The sections were washed in PBS to remove excess antibody and developed using the M.O.M. and peroxidase DAB kit, obtained from Vector Laboratories (Burlingame, CA). Sections were counterstained with hematoxylin, dehydrated, mounted in Permount Mounting Medium (Fisher Biotech) and photographed under Olympus BX41 bright field microscope. Hemotoxylin and eosin (H and E) pictures were photographed at 40X magnification. PCNA staining was photographed at 1000X magnification using oil. PCNA positive cells, which are the proliferating cells, were quantitated by counting 5 fields of 100 cells. Data is presented as the percentage of PCNA positive cells.

### siRNA Transfection and Assays

Chemically synthesized, double-stranded siRNA for E2F1, E2F2, E2F3, E2F4, E2F5 and E2F6 was purchased from Santa Cruz Biotechnology. The transfection experiments were performed in H69 and DMS114 cells [31], [45]. Asynchronous cells were harvested and re-plated in 96-well plates at about 40% confluence in growth media containing 10% FBS in the absence of antibiotics. The transfection of the above mentioned siRNA was performed by using Oligofectamine reagent (Invitrogen Corporation), according to the manufacturer's protocol. Eighteen hours post transfection, the cells were rendered quiescent for 36 hours by incubation in serum-free media. Subsequently, the cells were treated with 10% FBS in the presence of 50 µM capsaicin for 18 hours. The capsaicin was added 30 minutes prior to addition of the media containing 10% FBS. After 18 hours, the percentage of cells in S-phase was measured by the BrdU ELISA kit (Roche Laboratories). A non-targeting siRNA sequence (Santa Cruz Biotechnology) was used as a negative control for the transfection experiments. Each transfection was performed in duplicate, and the whole assay was repeated twice.

The results of the E2F4 transfection experiments were verified using a second set of independent siRNA obtained from Ambion Biotechnologies [31], [45]. The protocol of the transfection was same as previously described. A non-targeting control-siRNA was used as the negative control in all experiments. Each transfection was performed in duplicate, and the whole assay was repeated twice.

Western blotting experiments were performed to assess the expression of proteins after siRNA transfection [31], [45] in both H69 and DMS114 cells. Each transfection in the H69 cells was done using 5×10^5^ cells seeded in T-10 flasks (Midwest Scientific) in RPMI containing 10% FBS without antibiotics. In the case of the DMS114 cells, the transfection was performed in 6–well plates, using 5×10^5^ cells/well. In both the H69 cells and DMS114 cells, each transfection was performed in duplicate, and the whole experiment was repeated twice. The transfection of the indicated siRNA was performed using Oligofectamine reagent (Invitrogen Corporation), according to the manufacturer's protocol. A non-targeting control-siRNA was used as the negative control. After 36 hours of transfection, lysates were made as described above and the expression of E2F family of proteins was determined by western blotting analysis.

### Real-time PCR

H69 cells were subjected to serum starvation for 36 hours and subsequently stimulated with 10% FBS for 8 hours [46]. Total RNA was isolated using RNeasy miniprep kit from QIAGEN. One microgram of RNA was DNase treated using RQ1 DNase (Promega), followed by first-strand cDNA synthesis using the iScript cDNA synthesis kit (Bio-Rad). A fraction (1/20) of the final cDNA reaction volume was used in each PCR [46]. Primer sequences [46], [47] are as follows:

Cyclin E (forward primer): 5′TTCTTGAGCAACACCCTCTTCTGCAGCC3′.

Cyclin E (reverse primer): 5′TCGCCATATACCGGTCAAAGAAATCTTGTGCC3′.

TS (forward primer): 5′CTGCCAGCTGTACCAGAGAT3′.

TS (reverse primer): 5′ATGTGCATCTCCCAAAGTGT3′.

Cdc6 (forward primer): 5′CCCCATGATTGTGTTGGTAT3′.

Cdc6 (reverse primer): 5′TTCAACAGCTGTGGCTTACA3′.

18S (forward primer): 5′CTCAACACGGGAAACCTCAC3′.

18S (reverse primer): 5′AAATCGCTCCACCAACTAAGAA3′.

Real-time PCR was performed on a Bio-Rad iCycler.

### Chromatin Immunoprecipitation (ChIP) Assay

H69 were serum-starved for 36 hours by incubating them in serum-free RPMI-1640 [31], [32], [40], [48]. Subsequently, these quiescent H69 cells were re-stimulated with 10% FBS in the presence or absence of 50 µM capsaicin for 8 hours. Twenty-five million cells were used per IP reaction. Cells were treated with 1% formaldehyde for 10 minutes at room temperature to cross-link the proteins and the DNA. Cross-linking was terminated by the addition of 0.125 M glycine. Rabbit anti-mouse secondary antibody was used as the control for all reactions. PCR reactions were performed using 5 µL of DNA from the immunoprecipitation reactions or 1 µL of DNA from the input reaction as template. PCR cycling conditions for TS and cdc6 were as follows: 94°C for 2 min; then 35 cycles of 94°C for 30 s, 56°C for 30 s and 65°C for 30 s; followed by 65°C for 2 min. PCR cycling conditions for cyclin E were as follows: 94°C for 2 min; then 35 cycles of 94°C for 30 s, 66°C for 30 s and 72°C for 30 s; followed by 72°C for 2 min. The primer for cyclin E encompassed two of three E2F binding sites on the promoter as described in Nevins et al., (1994). These binding sites comprise the high affinity E2F binding sites on the human cyclin E promoter [49]. PCR for the c-Fos promoter (which is not regulated by E2F) was used as a negative control for all experiments. The sequences of the PCR primers used in the PCRs were as follows:

Cyclin E (forward primer): 5′CCCCGTCCCTGCGCCTCGCTG3′.

Cyclin E promoter (reverse primer): 5′CGGCGGCGGCGACGGCAGTGG3′.

Cdc6 promoter (forward primer): 5′GGCCTCACAGCGACTCTAAGA3′.

Cdc6 promoter (reverse primer): 5′CTCGGACTCACCACAAGC3′.

TS promoter (forward primer): 5′TGGCGCACGCTCTCTAGAGC3′.

TS promoter (reverse primer): 5′GACGGAGGCAGGCCAAGTG3′.

The cdc25A, c-fos primers and PCR conditions are described in [29].

### Statistical Analysis

All data was expressed as the mean ± SEM and represented using Graph Pad Prism 5. Differences between control and treated samples were analyzed by analysis of variance (ANOVA), followed by a Dunnett's multiple comparison test. The tumor growth rates in athymic mice were analyzed by ANOVA followed by Neuman-Keuls test. In the immunohistochemistry experiments, all PCNA positive nuclei were counted in five independent fields by two independent observers in a randomized double blind fashion. Chi-square analysis was done to determine differences in the percentage of PCNA positive nuclei. BrdU assays were performed in triplicate for each data point and the whole assay was repeated twice. PCNA ELISAs and caspase cleavage assays were performed in duplicate and the whole assay was repeated twice. All analyses were completed using a 95% confidence interval. Data was considered significant when the P value was less than 05.

## Results

### Capsaicin Displays Anti-proliferative Activity in Human SCLC Cells *in vitro*

**Figure 1:**
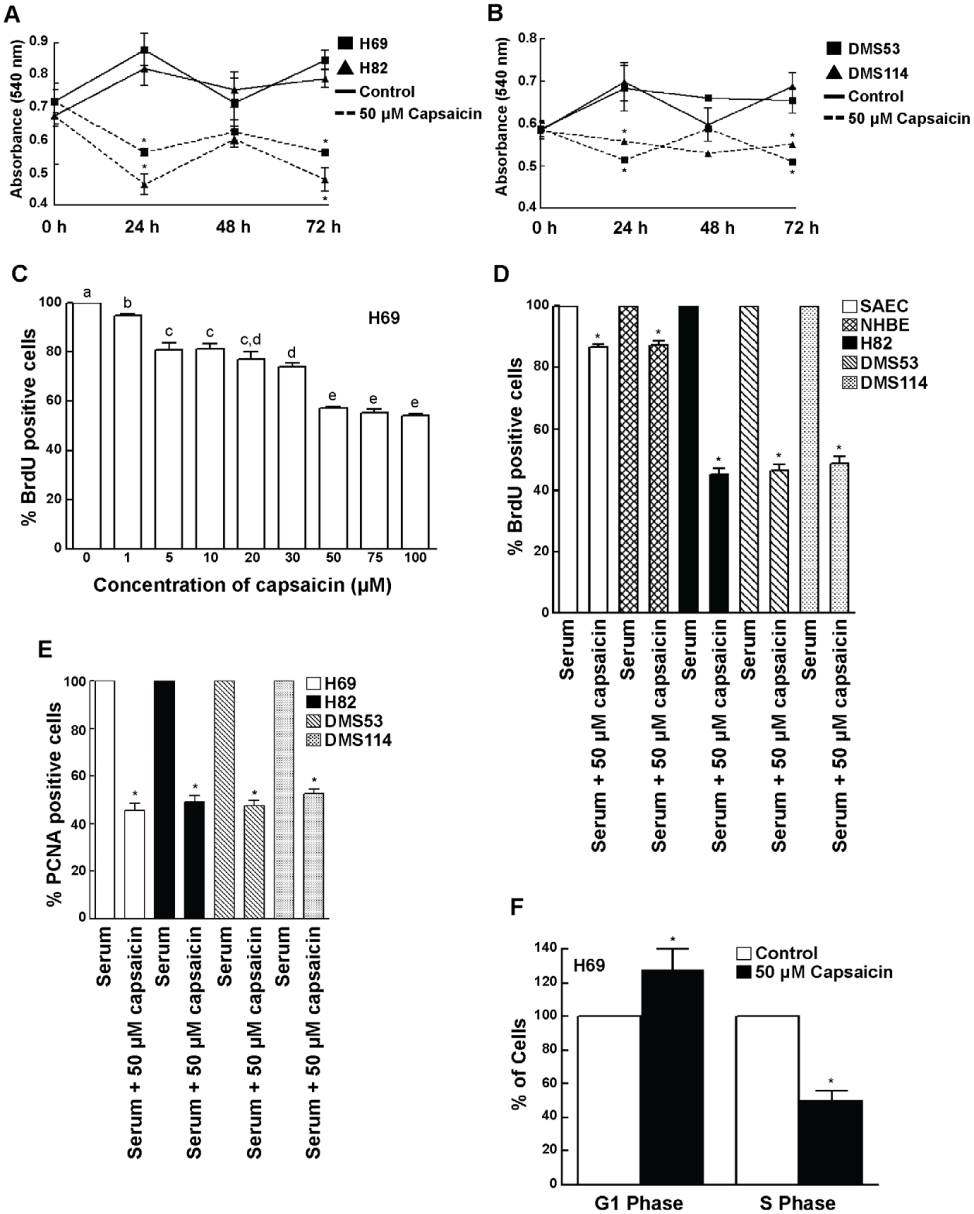
Capsaicin inhibited the proliferation of human SCLC cells in a time- and concentration-dependent manner. (A) MTT assays show at the treatment of H69 and H82 cells with 50 µM capsaicin causes a decrease in cell viability starting from 24 hours to 72 hours. (B) The MTT assay was repeated in DMS53 and DMS114 cells. Similar results were obtained. (C) Capsaicin displays potent dose-dependent anti-proliferative activity in H69 human SCLC cells. H69 cells were serum-starved for 36 hours and then re-stimulated with 10% FBS for 18 hours in the presence or absence of the indicated doses of capsaicin. BrdU incorporation assays were performed to assess the anti-proliferative effects of capsaicin. (D) The experiment was repeated in H82, DMS114 and DMS53 human SCLC cell lines and similar results were obtained. Most interestingly, capsaicin displayed relatively little anti-proliferative activity in SAEC and NHBE normal lung epithelial cells, indicating that the anti-proliferative activity of capsaicin was specific for lung cancer cells. (E) The results obtained from the BrdU assays were verified by the measurement of PCNA levels in human SCLC cells. Capsaicin decreased the number of PCNA positive cells in all the four SCLC cell lines. (F) FACS analysis confirms that capsaicin causes G1/S arrest in H69 human SCLC cells. H69 cells were serum-starved for 36 hours and subsequently re-stimulated with 10% FBS for 18 hours (to induce S-phase entry) in the presence or absence of 50 µM capsaicin. Cells were then stained with propidium iodide and analyzed by FACS to quantitate the relative percentage of cells in G1 and S phase. The percentage of cells in G1 and S-phase of untreated cells were taken to be 100%, and the relative percentages of capsaicin-treated cells were calculated relative to the control. Capsaicin increased the percentage of cells in G1 phase and concomitantly decreased the percentage of cells in S-phase, indicating the presence of G1/S arrest in capsaicin-treated H69 cells. Values indicated by an “*” or a different letter are statistically significant.

MTT assays were performed to determine whether capsaicin can suppress the growth of human SCLC cells *in vitro* [37]. Treatment of H69 and H82 human SCLC cells with 50 µM capsaicin can potently suppress the growth of these cells in a time dependent manner, with the maximal growth-inhibitory effect of capsaicin being displayed at 72 hours post treatment (Fig. 1, A). We repeated the experiment in DMS53 and DMS114 human SCLC cells and obtained similar results (Fig. 1, B).

The next question we asked was whether capsaicin could cause inhibition of cell proliferation in human SCLC cells. BrdU assays were used to examine the anti-proliferative effects of capsaicin in four human SCLC cell lines, namely H69, H82, DMS53 and DMS114 [31], [48] and two normal lung epithelial cell lines (NHBE and SAEC). Serum-starved H69 cells were re-stimulated with 10% FBS in the presence or absence of capsaicin. BrdU is a thymidine nucleotide analog which is incorporated (instead of thymidine) only into the replicating DNA of proliferating S-phase cells. The amount of incorporation is measured by ELISA technique. The absorbance of cells treated with 10% FBS was assumed to be 100%, and capsaicin-induced decrease in S-phase was calculated as a percentage of the 10% FBS treated cells.

Capsaicin inhibited the proliferation of H69 human SCLC cells in a concentration dependent manner (Fig. 1, C). The anti-proliferative activity of capsaicin was maximal at 50 µM and remained constant thereafter until 100 µM. Therefore, the concentration of 50 µM capsaicin was used for all subsequent experiments. BrdU assays showed that 50 µM capsaicin displayed relatively little anti-proliferative activity in both NHBE and SAECs, whereas it displayed robust growth-inhibitory activity in the human SCLC cell lines at the same concentrations (Fig. 1, D). We believe these results constitute an important finding; capsaicin suppresses cell growth in lung cancer cells and minimally affects normal lung cells.

The results of the BrdU assays were further verified by measurement of the PCNA levels in human SCLC cells treated with capsaicin. Quiescent human SCLC cells were stimulated with serum for 18 hours (to induce S-phase entry) in the presence or absence of 50 µM capsaicin. After 18 hours, PCNA levels were measured by ELISA. PCNA ELISA assays showed that treatment of H69, H82, DMS53 and DMS114 human SCLC cells with 50 µM capsaicin resulted in about a 50% inhibition of S-phase entry of cells (Fig. 1, E).

Finally, we performed cell cycle analysis by flow cytometry to confirm the results obtained from the BrdU and PCNA experiments [15], [39]. Serum-starved H69 cells were re-stimulated with 10% FBS in the presence or absence of 50 µM capsaicin. Subsequently, the cells were fixed with ethanol, stained with propidium iodide and analyzed by flow cytometry. The percentage of cells in the G1-phase and S-phase of untreated cells were taken to be 100%, and the relative distribution of cells in G1- and S-phase of capsaicin-treated cells were calculated as a percentage of the control untreated cells (Fig. 1, F). Fifty micromole capsaicin caused an increase in the fraction of G1 cells and a concomitant decrease in the percentage of H69 cells in S-phase (Fig. 1, F). Taken together, our results show for the first time that capsaicin displays potent anti-proliferative activity in human SCLC cells *in vitro*.

### Anti-proliferative Effect of Capsaicin Was Correlated with Decrease of E2F S-phase Genes

**Figure 2:**
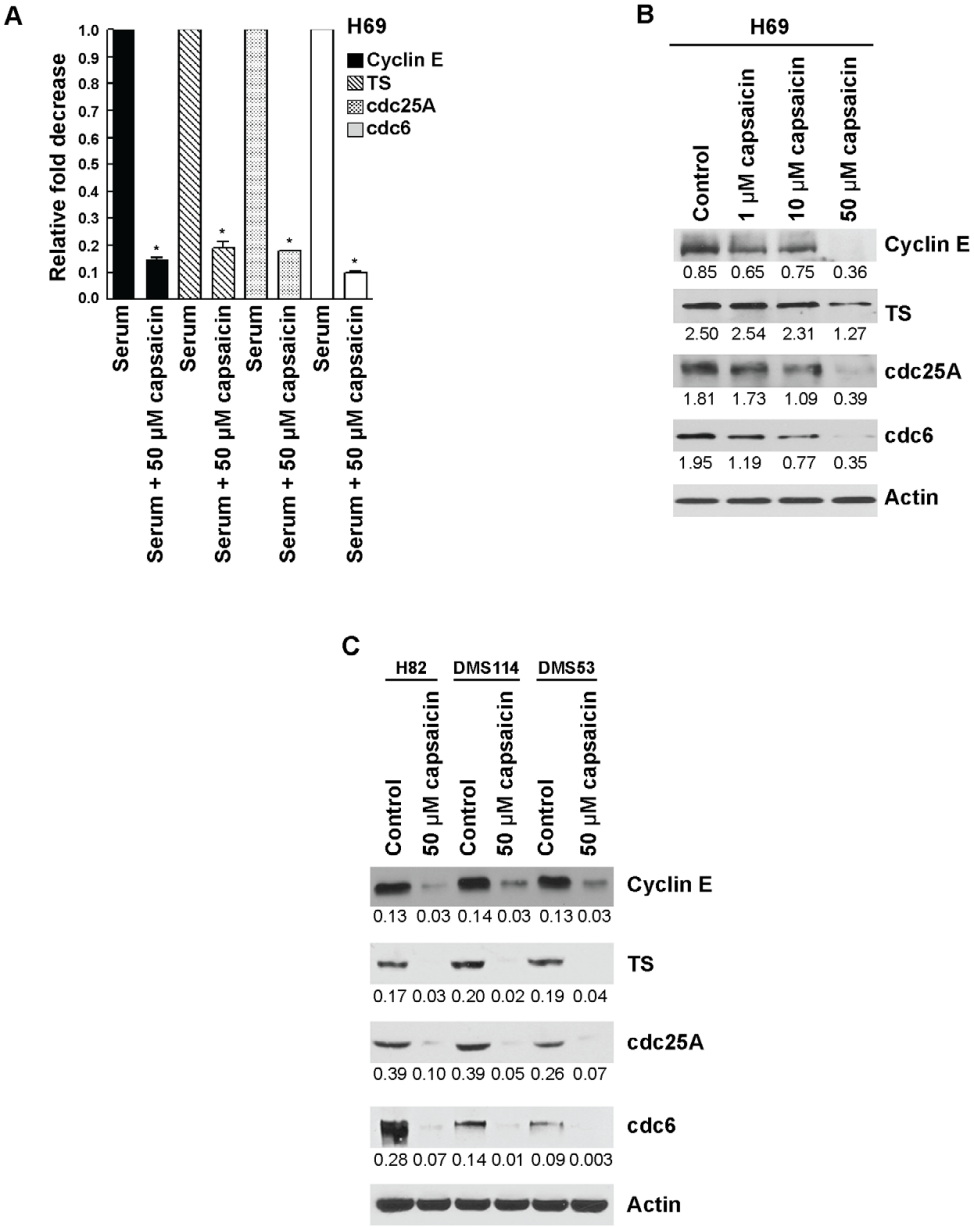
The treatment with capsaicin correlated with decreased expression of E2F-responsive proliferative S-phase genes. (A) Real-time PCR analysis indicated that capsaicin decreased the mRNA levels of cyclin E, TS, cdc25A and cdc6. (B) Western blotting analysis showed that capsaicin caused a concentration-dependent decrease in the levels of cyclin E, TS, cdc25A and cdc6 in H69 human SCLC cells. β-actin was used as the loading control for the western blotting experiments and the results were quantitated by densitometric analysis. (C) The western blotting experiment was repeated in H82, DMS53 and DMS114 human SCLC cells treated with 50 µM capsaicin and similar results were observed. Values indicated by an “*” are statistically significant.

Next, we wanted to examine whether capsaicin-induced growth arrest was correlated with any changes in the expression of E2F-target proliferative genes. We selected cyclin E, TS, cdc25A and cdc6. Since these are E2F target genes, we examined whether capsaicin could decrease the mRNA level by quantitative real-time-PCR using RNA extracted from serum-stimulated cells and cells treated with serum and 50 µM capsaicin. Our results showed that serum-stimulated expression of cyclin E, TS, cdc25A, and cdc6 was significantly down-regulated by 50 µM capsaicin (P<01; Fig. 2, A). These results were confirmed by western blotting analysis. Capsaicin suppressed the protein levels of all the above genes in a concentration dependent manner in H69 human SCLC cells (Fig. 2, B). We repeated the western blotting experiments in H82, DMS53 and DMS114 cells treated with 50 µM capsaicin and obtained similar results (Fig. 2, C). Taken together, capsaicin suppressed the expression of cyclin E, TS, cdc25A and cdc6 both at mRNA and protein levels in human SCLCs.

### Capsaicin Inhibited the Growth of Human H69 Cells *in vivo*

**Figure 3:**
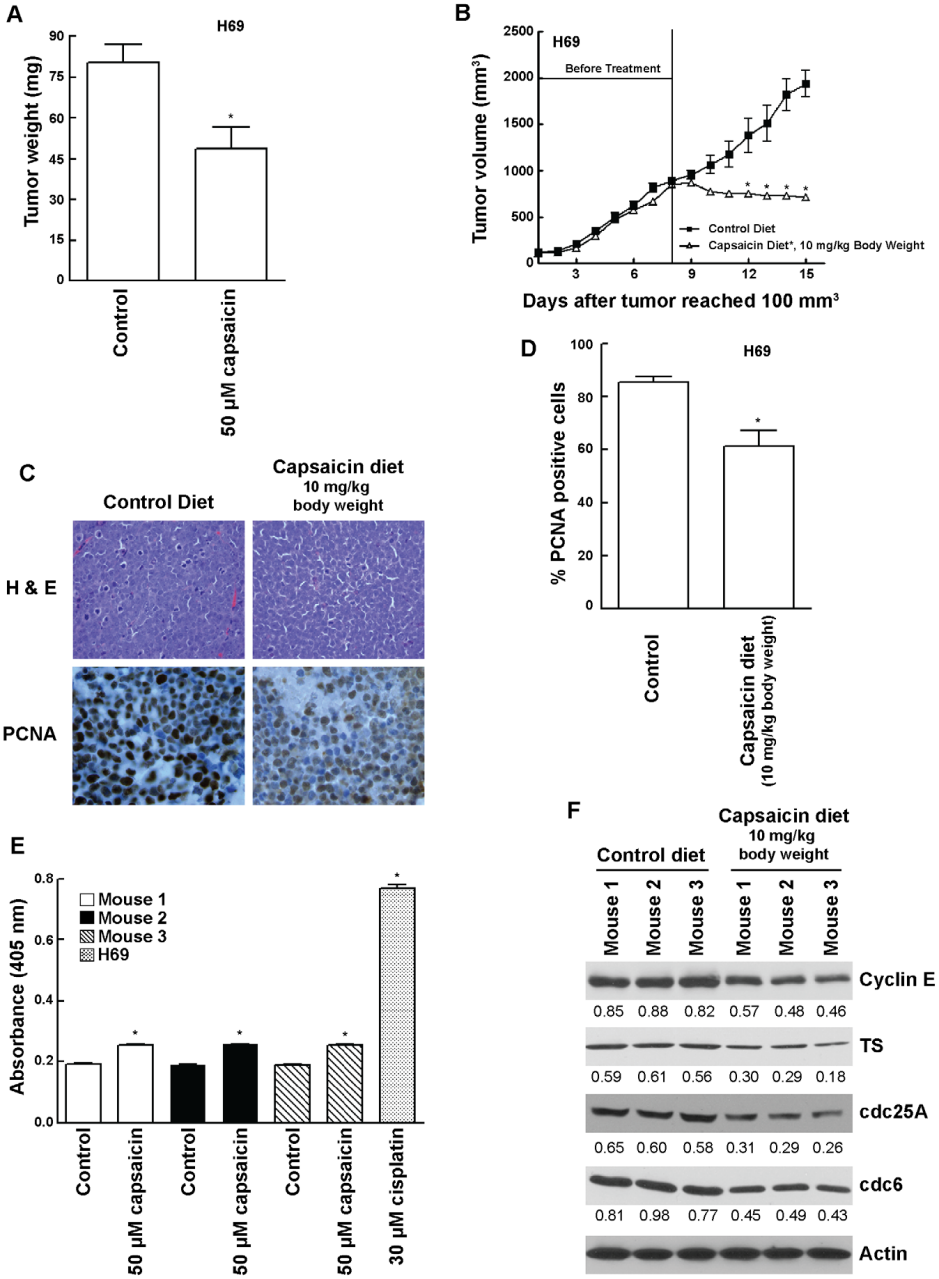
Capsaicin inhibited the growth of human SCLC tumors *in vivo*. (A) Chicken chorioallantoic membrane (CAM) assays showed that 50 µM capsaicin suppressed the growth of H69 tumors on chicken CAM. (B) Capsaicin suppressed the growth of established SCLC tumors in nude mice models. H69 cells were injected subcutaneously between the scapulae of nude mice. After the tumors attained a threshold volume of 100 mm^3^, the tumors were allowed to grow until a volume of 800 mm^3^, after which animals were divided into two groups. The treatment group was administered 10 mg of capsaicin/kg body weight in an AIN-76A based diet. The control group was administered with an AIN-76A based diet containing 10% corn oil (vehicle for capsaicin) only. Tumor volumes were calculated as (l X w X h)/2. (C) Tumor sections were stained with H and E (top panels) to assess cellular morphology and immunostained for PCNA to assess cell proliferation (bottom panels). Nude mice treated with 10 mg capsaicin/kg body weight displayed reduced cell proliferation as evidenced by decreased PCNA staining (bottom right panel) relative to control (bottom left panel). (D) Quantitation of PCNA positive cells indicated that the administration of capsaicin reduced cell proliferation in H69 tumors, relative to controls. (E) The apoptotic activity of capsaicin in nude mice models was measured by caspase cleavage assays of mouse tumor lysates. Tumor lysates from capsaicin treated mice displayed only a little increase in cellular apoptosis as compared to control mice. H69 lysates treated with 30 µM cisplatin for 72 hours was taken as the positive control for the assay. (F) Western blotting of tumor lysates from mice showed that capsaicin treatment decreased the expression of E2F-responsive proliferative genes namely cyclin E, TS, cdc25A and cdc6. β-actin was used as the loading control for the western blotting experiments. The results were quantitated by densitometric analysis. Values indicated by an “*” are statistically significant.

Previous studies have shown that human cancer cells implanted on chicken chorioallantoic membrane (CAM) constitute an established model to study tumor growth *in vivo* [41]. The administration of 50 µM capsaicin significantly attenuated (P = 02) the growth of H69 human SCLC tumors implanted on CAM membrane (Fig. 3, A). The results of these experiments were confirmed by using nude mice models. The administration of capsaicin (10 mg/kg body weight) significantly reduced the growth rate of established (800 mm^3^) H69 tumors xenotransplanted in nude mice (Fig. 3, B).

Tumors were harvested at the end of the treatment. Sections were stained with H and E and analyzed by immunohistochemical staining with monoclonal antibody to PCNA (Fig. 3, C). There was a significant reduction of PCNA positive cells (P<001) in viable areas of H69 tumors removed from in nude mice that consumed capsaicin (Fig. 3, C). The quantitation of PCNA staining in tumors confirms a significant decrease in proliferation of cells treated with capsaicin (P<01; Fig. 3, D). We also wanted to assess whether capsaicin displayed anti-apoptotic activity in human SCLC tumor-bearing nude mice. Caspase cleavage of mouse tumor lysates showed that capsaicin treated mice displayed very little increase in apoptotic activity as compared to control mice (Fig. 3, E). H69 lysates treated with 30 µM cisplatin for 72 hours were used as the positive controls for the assay. Therefore, we conjectured that perhaps capsaicin was inhibiting the growth of H69 tumors primarily by causing cell cycle arrest. Western blotting analysis of tumor lysates showed that H69 tumors isolated from capsaicin treated mice had lower levels of cyclin E, TS, cdc25A and cdc6, as compared to control untreated mice (Fig. 3, F). These observations seemed to suggest that the anti-proliferative effect of capsaicin was correlated with a decrease in levels of E2F responsive S-phase genes *in vivo*.

### Effect of E2F-siRNAs on the Anti-proliferative Activity of Capsaicin

In the next series of experiments, the effect of E2F1–6 in the anti-proliferative activity of capsaicin was assessed by siRNA methodology. E2F1, E2F2, E2F3, E2F4, E2F5 and E2F6 siRNAs were individually transfected in H69 cells. Eighteen hours after transfection, H69 cells were serum-starved for 36 hours (by incubating them in serum-free RPMI) and subsequently re-stimulated with 10% FBS in the presence of 50 µM capsaicin for 18 hours. BrdU assays were performed to measure the percentage of cells undergoing S-phase entry [31].

**Figure 4:**
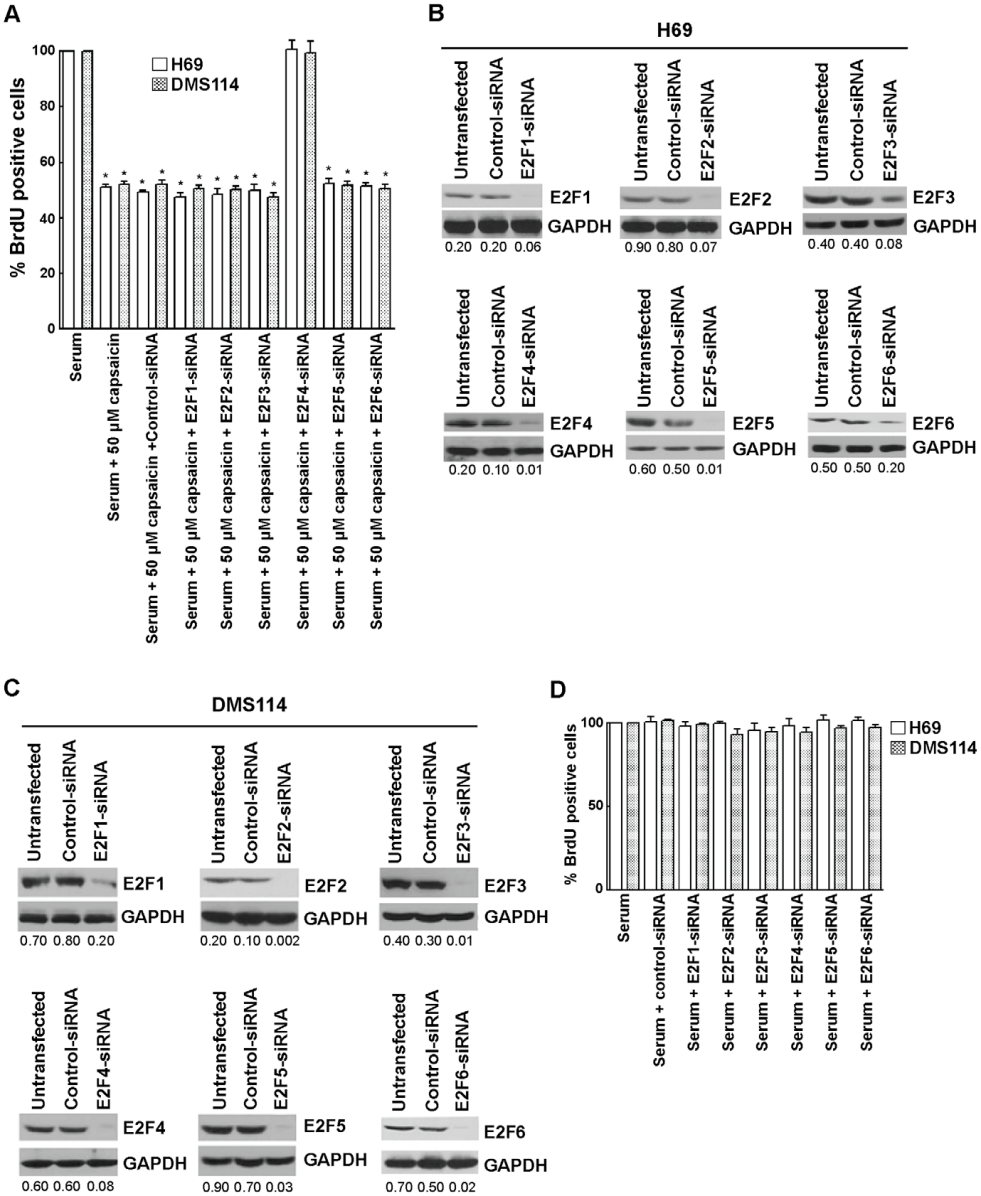
BrdU assays demonstrated that depletion of E2F4 ablates the anti-proliferative activity of capsaicin. (A) H69 and DMS114 cells were transfected with the indicated E2F-siRNA as detailed in “Materials and Methods.” Eighteen hours post transfection, the cells were serum-starved for 36 hours and re-stimulated with media containing 10% FBS in the presence of 50 µM capsaicin for 18 hours. Subsequently, BrdU assays were performed to measure cell proliferation. The anti-proliferative activity of capsaicin was ablated by E2F4-siRNA but not affected by a non-targeting control-siRNA. (B) Western blotting analysis confirmed the suppression of E2F1–6 expression upon siRNA transfection. GAPDH was used as the loading control for the western blotting experiments, and the results were quantitated by densitometric analysis. (C) Western blotting experiments demonstrated that levels of E2F1–6 were decreased upon siRNA transfection in DMS114 cells. (D) BrdU assay showed that transfection of E2F1–6 siRNA without capsaicin did not affect the proliferation of cells in response to 10% FBS. This indicates that the effects of E2F-siRNAs observed in (A) are specifically mediated by capsaicin treatment. Values indicated by an “*” are statistically significant.

We observed that E2F4-siRNA reversed the anti-proliferative effect of capsaicin in H69 and DMS114 cells, whereas E2F1–3-, E2F5- and E2F6-siRNA did not have any effect on the growth-inhibitory effects of capsaicin (Fig. 4, A). The control non-targeting siRNAs did not have any effect on the anti-proliferative effect of capsaicin (Fig. 4, A). Western blotting analysis showed that the transfection of the above mentioned siRNAs suppressed E2F1–6 protein levels in both H69 and DMS114 cells (Fig. 4, B and C, respectively).

Since the E2F-family of transcription factors have been shown to play a pivotal role in cell proliferation, we wanted to examine whether E2F-siRNAs would affect proliferation of human SCLC cells untreated with capsaicin. E2F1–6-siRNAs were individually transfected in H69 cells using Oligofectamine reagent. Eighteen hours post-transfection, H69 cells were serum-starved for 36 hours (by incubating them in serum-free RPMI) and subsequently re-stimulated with 10% FBS for 18 hours. BrdU assays were performed to measure the percentage of cells undergoing S-phase entry [31]. We found that none of the E2F-siRNAs individually affected the proliferation of SCLC cells stimulated with 10% FBS (Fig. 4, D). The experiment was repeated in a second SCLC cell line, DMS114, and similar results were observed (Fig. 4, D). Taken together, our results show that E2F4-siRNA specifically abrogates the anti-proliferative effect of capsaicin and does not affect serum-induced proliferation of SCLC cells.

**Figure 5:**
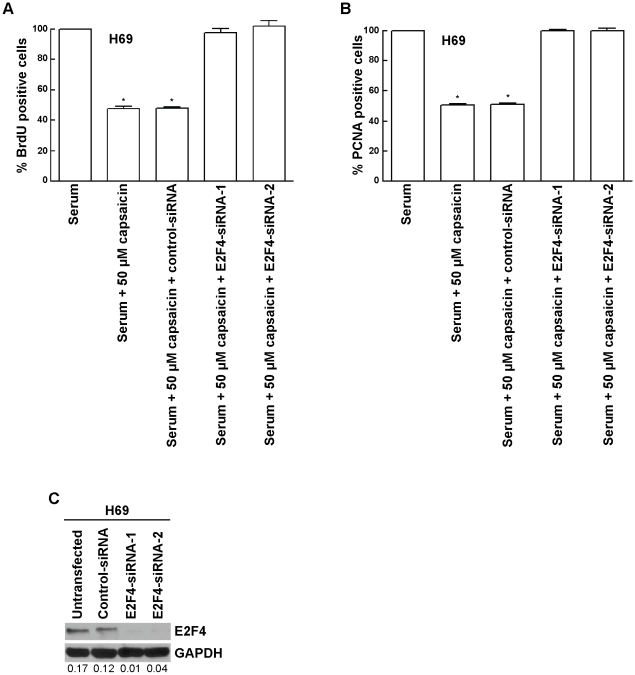
Two independent E2F4-siRNA reversed the anti-proliferative effect of capsaicin in H69 cells. (A) The transfection of siRNA and subsequent BrdU assay was performed as described in “Materials and Methods.” (B) The results obtained by BrdU assay are confirmed with PCNA ELISA assay. Two independent E2F4-siRNA were transfected as described in “Materials and Methods.” PCNA assays were then performed to assess the levels of E2F4-siRNA on the anti-proliferative activity of capsaicin. (C) Western blotting analysis indicated that E2F4 levels are suppressed upon siRNA transfection. GAPDH was used as the loading control for the western blotting experiments, and the results were quantitated by densitometric analysis. Values indicated by an “*” are statistically significant.

The results of the siRNA experiments were verified by using two independent sets of E2F-siRNAs. BrdU assays showed that both independent E2F4-siRNA ablated the effects of 50 µM capsaicin in H69 cells (Fig. 5, A), whereas a non-targeting control-siRNA did not have any effect. The data obtained from the BrdU assays was further verified by PCNA-ELISA assays, and similar results were obtained (Fig. 5, B). Western blotting analysis showed that the transfection of either sets of E2F4-siRNAs produced efficient suppression of E2F4 protein levels in H69 cells (Fig. 5, C).

### Capsaicin Promotes the Recruitment of E2F4 to E2F-responsive Proliferative Promoters

Our data seemed to indicate that E2F4 plays a vital role in the anti-proliferative effects of capsaicin. Therefore, ChIP assays were performed to examine the differential occupancy of E2F family of proteins on E2F-responsive proliferative promoters (cyclin E, TS, cdc25A and cdc6) upon capsaicin-treatment of H69 human SCLC cells [31], [32], [40], [45].

**Figure 6:**
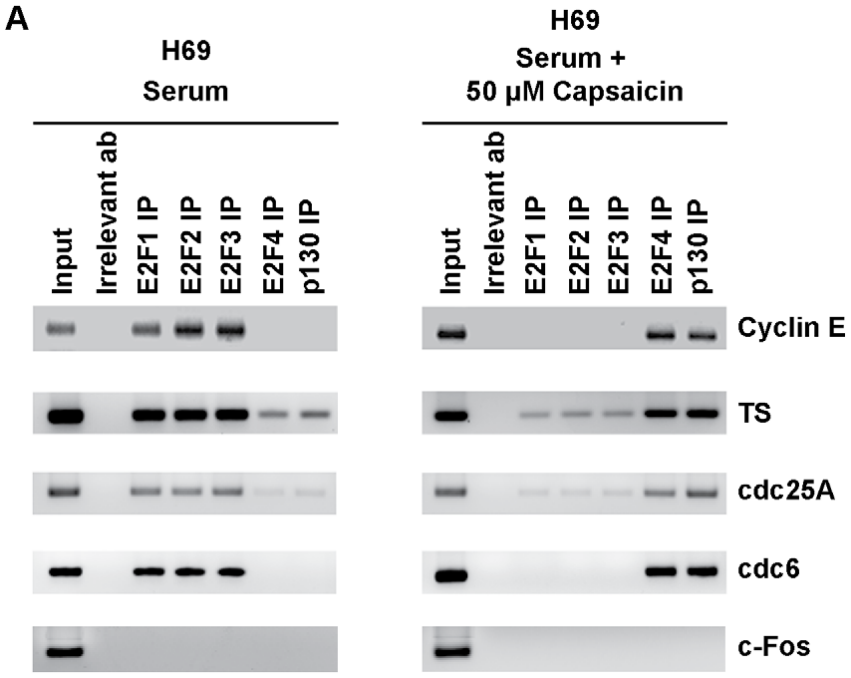
ChIP assays showed that capsaicin induces the recruitment of E2F4 and p130 on proliferative promoters. Serum-stimulated H69 cells contained robust amounts of proliferative E2Fs, namely E2F1, E2F2 and E2F3, associated with E2F-responsive proliferative promoters like cyclin E, TS, cdc25A and cdc6 (left panel). The treatment of H69 cells with 50 µM capsaicin causes dissociation of proliferative E2Fs and recruitment of the repressive E2F4 and p130 on these promoters (right panel). PCR for the c-Fos promoter (which is not regulated by E2F) was taken as the control for the experiment.

High amounts of the proliferative E2Fs, namely E2F1, E2F2 and E2F3, were bound to all E2F-responsive promoters in the control serum-stimulated H69 cells (Fig. 6, left panel, lanes 3,4,5 from the left). In contrast, no E2F4 or very low levels of E2F4 were bound to these promoters in proliferating H69 cells (Fig. 6, left panel, lane 6 from left). However, the treatment of these cells with 50 µM capsaicin for 8 hours resulted in the recruitment of robust amounts of E2F4 on cyclin E, TS, cdc25A and cdc6 promoters (Fig. 6, right panel, lane 6 from left), accompanied with concomitant dissociation of E2F1, E2F2 and E2F3 (Fig. 6, right panel, lane 3,4,5 from left). Furthermore, the enhanced binding of E2F4 was accompanied by recruitment of the E2F4-binding partner p130 on the cyclin E, TS, cdc25A and cdc6 promoters in capsaicin-treated cells. In contrast, very low levels of p130 were bound to these promoters in proliferating cells (Fig. 6, left panel, last lane from left). Therefore, our data seem to suggest that capsaicin selectively recruits E2F4 and p130 to proliferative promoters, thereby repressing their transcription and inhibiting S-phase entry in human SCLC cells. PCR for the c-Fos promoter (which is not regulated by E2F) was used as a negative control for the ChIP experiments. We observed that there were no E2F family proteins associated with this promoter (Fig. 6, bottom lanes).

## Discussion

The dysfunction of the E2F/Rb pathway is a hallmark of greater than 90% of lung cancers [3], [4], [5]. Several convergent studies have indicated that the majority of human SCLC contains mutations/deletions in vital tumor suppressor genes like Rb, p130, p16, cyclin D1 and p53, which facilitate its growth and distant metastasis [3]. Therefore, it is probable that the anti-proliferative activity of nutritional agents like capsaicin is mediated by its effects on the cell cycle machinery in human SCLC cells.

Emerging evidence shows that capsaicin is a promising anti-cancer agent that causes potent apoptosis in prostate cancer, non small cell lung cancer, gliomas and gastric cancers [10], [13], [14], [15], [16], [21], [50]. However, the growth-inhibitory activity of capsaicin in human SCLC is unknown. To our knowledge, our pilot study showed for the first time that capsaicin displays potent anti-proliferative activity in human SCLCs both in cell culture models and in two *in vivo* models. Our results showed that capsaicin suppresses the growth of established H69 human SCLC tumors in both CAM and nude mice models. We observed that the administration of capsaicin did not cause any gross discomfort, toxicity or weight loss in these animals. Taken together, our data suggest that capsaicin has the potential for therapy and management of human SCLCs.

The majority of studies exploring the anti-cancer activity of capsaicin have focused on the mechanisms underlying capsaicin-induced apoptosis [10], [13], [14], [15], [16], [21], [50]. Only a few studies have investigated the signaling pathways underlying capsaicin-induced cell cycle arrest [10], [17], [19], [21]. Our results showed for the first time that capsaicin induces G1 arrest in four human SCLC cell lines in a concentration dependent manner. Our data are in agreement with results of previous studies, which have shown that capsaicin induces G1 arrest in prostate cancer, breast cancer, epidermoid cancer and human leukemic cells [10], [17], [19], [21]. Min et al., (2004) have found that capsaicin can inhibit the proliferation of endothelial cells and displays anti-angiogenic activity in both cell culture and mouse models [22]. Similarly, capsaicin analogs like capsiate and dihydrocapsiate have been found to inhibit VEGF-induced angiogenesis [51]. It is probable that the anti-angiogenic activity of capsaicin is, at least in part, responsible for its observed anti-tumor activity in mice models and the CAM model. We quantified the extent of angiogenesis observed in our CAM experiments and found that the capsaicin-treated H69 tumor-bearing CAM contained fewer blood vessels (3.4±0.6) than those of the untreated controls (8.5±0.9). Other mechanisms of the anti-proliferative activity of capsaicin included regulation of cyclin/cdks, p21 and p53 in the aforementioned cells. However, no studies have explored the role of downstream E2F family of proteins in the anti-proliferative effects of capsaicin.

One of the interesting findings of our study was that E2F1-, E2F2- and E2F3-siRNA by themselves had no effect on the serum-induced proliferation of H69 cells. This observation can be explained by the fact that there is considerable redundancy in the proliferative functions of the E2F family of proteins [25], [27], [28], [29], [30]. E2F1–3 are proliferative E2Fs. Therefore, if one of them, for example E2F1, is knocked down by E2F1-siRNA, it is probable that its mitogenic functions can be compensated by other E2F2 and E2F3 [25], [27], [28], [29], [30]. Thus, no effect of these siRNAs on serum-induced proliferation of H69 cells is observed. E2F4, E2F5 and E2F6 are repressive E2F proteins; therefore, their siRNAs would not be expected to have an impact on cell proliferation.

Our study demonstrated for the first time that E2F4 primarily mediates the anti-proliferative activity of capsaicin. Our data is consistent with previous studies that have shown that E2F4 is a repressor of transcription and inhibits cell proliferation. In addition, the E2F4/p130 pathway has been implicated in the growth and progression of lung cancer. E2F4−/− mice were found to have defects in small airway epithelial cells, suggesting a role for this protein in lung development [52]. Studies from E2F4 (−/−) Rb (−/−) chimeric mice have suggested that E2F4 may play a role in early stages of small cell lung cancer [53]. Bankovic et al., (2009) studied genomic instability in NSCLC patients by DNA fingerprinting and discovered that E2F4 was among the group of genes responsible for growth and metastasis of NSCLCs [33]. Ren et al., (2002) performed a ChIP analysis combined with microarray experiments to determine transcriptional targets of E2F4. Their results indicated that E2F4 target genes include those regulating DNA damage checkpoint, DNA repair, mitotic spindle checkpoint and chromatin assembly/condensation [54]. Many of these processes are involved in neoplastic transformation. Similarly, the E2F4 binding pocket protein, p130, has been suggested as a novel molecular target for diagnosis and therapy of lung cancers [55], [56]. Gene therapy studies have shown that over expression of p130 in advanced stage lung tumors could attenuate their growth [55], [57]. Furthermore, p130 is involved in tumor angiogenesis and plays a vital role in the differentiation and mobilization of bone marrow-derived endothelial cell precursors and endothelial sprouting from neighboring vessels [58]. We believe that nutritional agents like capsaicin recruit the E2F4/p130 pathway to exert anti-proliferative effects in human SCLCs.

ChIP assays were performed to examine the relative distribution of E2Fs on E2F-responsive promoters in human SCLC cells. Our studies revealed that the treatment of human SCLC cells with capsaicin leads to differential recruitment of E2Fs on E2F-responsive promoters like cyclin E, TS, cdc25A and cdc6. Control H69 SCLC cells contained E2F1, E2F2 and E2F3 bound to proliferative promoters. Our results are consistent with previous studies in that proliferative responses are primarily mediated by E2F1, E2F2 and E2F3, whereas the recruitment of E2F4 on the promoters suppresses cell proliferation [31], [32], [38]. The treatment of H69 human SCLC cells with capsaicin leads to a switch in E2F subtypes on cyclin E, TS, cdc25A and cdc6 promoters; E2F1–3 are dissociated from the promoter, and E2F4 and p130 are recruited. We did not detect any E2F5 on any of the promoters (data not shown).

In summary, the data presented in this paper show that capsaicin displays potent anti-proliferative activity against human SCLC, and this effect is mediated by the E2F4 pathway. Aberrancies in the E2F pathway are one of the hallmarks of human SCLC [3], [4], [30], [57]; therefore, nutritional agents like capsaicin, which target the E2F pathway, may represent new avenues for the treatment of lethal malignancies like small cell lung cancer. We believe that the data obtained from this pilot study establishes the “proof–of-principle” for these concepts. Future research will focus on the identification of second generation capsaicin-mimetics and the exploration of their anti-proliferative activity and signaling pathways in human SCLCs.
